# Function of Golgi-centrosome proximity in RPE-1 cells

**DOI:** 10.1371/journal.pone.0215215

**Published:** 2019-04-15

**Authors:** Kati Tormanen, Celine Ton, Barbara M. Waring, Kevin Wang, Christine Sütterlin

**Affiliations:** Department of Developmental and Cell Biology, University of California, Irvine, Irvine, California, United States of America; Institut de Genetique et Developpement de Rennes, FRANCE

## Abstract

The close physical proximity between the Golgi and the centrosome is a unique feature of mammalian cells that has baffled scientists for years. Several knockdown and overexpression studies have linked the spatial relationship between these two organelles to the control of directional protein transport, directional migration, ciliogenesis and mitotic entry. However, most of these conditions have not only separated these two organelles, but also caused extensive fragmentation of the Golgi, making it difficult to dissect the specific contribution of Golgi-centrosome proximity. In this study, we present our results with stable retinal pigment epithelial (RPE-1) cell lines in which GM130 was knocked out using a CRISPR/Cas9 approach. While Golgi and centrosome organization appeared mostly intact in cells lacking GM130, there was a clear separation of these organelles from each other. We show that GM130 may control Golgi-centrosome proximity by anchoring AKAP450 to the Golgi. We also provide evidence that the physical proximity between these two organelles is dispensable for protein transport, cell migration, and ciliogenesis. These results suggest that Golgi-centrosome proximity *per se* is not necessary for the normal function of RPE-1 cells.

## Introduction

The close physical proximity between the Golgi and the centrosome is a typical feature of mammalian cells. In these cells, Golgi membranes are organized as an interconnected ribbon in the perinuclear region of a cell, adjacent to the centrosome, the major microtubule organizing center. This proximity is unique to mammalian cells and not found in yeast, plant or fly cells [1,2]. The molecular mechanisms that establish and maintain Golgi-centrosome proximity and its functional significance remain incompletely understood.

Golgi-centrosome proximity is disrupted by conditions that induce loss of Golgi organization. These include drug-induced Golgi fragmentation, as seen for example with nocodazole, which depolymerizes microtubules, or illimaquinone, which induces Golgi vesiculation [3–5]. Golgi fragmentation and the resulting separation of Golgi and centrosome is also observed upon depletion of structural Golgi proteins, such as Golgin-84, Golgin-160 or GMAP210, [6–8]. Finally, Golgi membranes are completely fragmented and dispersed during mitosis (reviewed in [9,10]).

Effects on Golgi-centrosome proximity have also been reported for the depletion of TBCCD1, a centrosome-associated protein that is related to tubulin co-factor C protein [11]. TBCCD1-depleted cells displayed fragmented and dispersed Golgi membranes. In addition to this pronounced Golgi phenotype, the centrosome lost its typical perinuclear position and was mislocalized to the cell periphery. As a result, microtubules were disorganized, and there were defects in cell migration and primary cilia formation.

Only few conditions have been found to alter Golgi-centrosome proximity without majorly affecting Golgi organization. Expression of an N-terminal domain of AKAP450, a large Golgi and centrosome-localized scaffolding protein, induced separation of Golgi and centrosome [12]. This domain associated with the p150^Glued^ subunit of the dynactin complex and the Golgi protein GM130. In these cells, Golgi morphology and global protein secretion were fairly normal. However, there was a defect in directional protein transport, cell migration and ciliogenesis [12].

Several studies suggest a possible role for GM130 in the control of Golgi-centrosome proximity. GM130 is a peripheral protein of the *cis*-Golgi. This well- studied member of the golgin family of proteins interacts with AKAP450, and is proposed to function as a scaffolding protein [13]. When unpolarized osteosarcoma (U2OS) cells were depleted of GM130 by RNAi, Golgi membranes retained their stacked, perinuclear appearance. However, there were defects in centrosome organization and positioning, which resulted in microtubule disorganization and a block in cell migration [14]. Furthermore, in Purkinje neurons and mouse embryonic fibroblasts from GM130 knockout mice, Golgi and centrosome were physically separated, and directional protein transport was disrupted [15]. To date, it is not understood how GM130 helps with establishing the link between Golgi and centrosome.

In the present study, we examined how and why Golgi and centrosome are in close physical proximity in retinal pigment epithelial cells (RPE-1). We used CRISPR/Cas9-mediated gene editing to generate two clonal GM130 knockout cell lines. In these cell lines, Golgi and centrosome were separated, but protein transport, cell migration and ciliogenesis were unaffected. These results suggest that at least in RPE-1 cells, Golgi-centrosome proximity is dispensable for cell homeostasis.

## Materials and methods

### Cell culture and treatments

hTERT-RPE-1 (ATCC) cells were maintained in DMEM (GIBCO) supplemented with 10% FBS (HyClone) in a 37°C incubator with 5% CO_2_. For cilia formation assays, cells were incubated in DMEM without serum for 48 hours.

### CRISPR-Cas9

GM130 knockout (KO) cell lines were established with the Alt-R CRISPR-Cas9 System following the manufacturer’s instructions (Integrated DNA Technologies), using the guide RNAs listed below. Clone 60 (KO60) was generated by targeting exon 1 with a single gRNA, while clone 2 (KO2) was generated through the use of four guide RNAs.

The guide RNA sequences are listed in Table 1.

**Table 1:** GM130 specific gRNA sequences.

Target	Sequence, 5'/AltR1/ to 3'/AltR2/
GM130 Exon 1	rGrGrG rUrUrU rCrUrU rCrCrG rArCrA rUrCrG rCrGrG rUrUrU rUrArG rArGrC rUrArU rGrCrU
GM130 Intron 3–4	rArCrA rUrGrC rUrArG rGrArG rCrCrA rCrUrC rArGrG rUrUrU rUrArG rArGrC rUrArU
GM130 Intron 3–4	rArArC rCrArA rUrCrC rArCrA rCrCrC rCrUrG rArGrG rUrUrU rUrArG rArGrC rUrArU rGrCrU
GM130 Intron 4–5	rUrCrG rGrUrU rGrArU rGrArG rArArA rGrUrC rCrUrG rUrUrU rUrArG rArGrC rUrArU rGrCrU
GM130 Intron 4–5	rCrUrG rUrUrG rGrGrG rCrArG rCrArG rUrCrU rCrUrG rUrUrU rUrArG rArGrC rUrArU rGrCrU

In brief, each gRNA was annealed to a CRISPR-Cas9 tracrRNA at equimolar ratios, incubated at 95 °C for 5 minutes and allowed to cool to room temperature. This RNA complex was then mixed with the Cas9 enzyme at equimolar ratios. Lipofectamine RNAiMax Transfection Reagent (2μl, Invitrogen) was used to deliver 10nM of the ribonucleoprotein complexes to 4x10^4^ RPE cells in a 96 well plate using reverse transfection. 48 hours after transfection, cells were plated for individual clones. Approximately 130 clones were screened by immunofluorescence, and two clones were selected for further verification by western blotting and genomic PCR followed by sequencing (Retrogen).

### Immunofluorescence microscopy

Cells were grown on glass coverslips and fixed with ice-cold methanol (JT Baker) for 7 minutes or with 4% paraformaldehyde (TedPella) for 10 minutes. Cells were blocked and permeabilized with 2% blocking buffer (2% FBS, 0.01% TritonX-100, and 1 X PBs), stained with primary antibodies for 1 hour at room temperature followed by staining with secondary antibodies (Lifetechnologies) for 1 hour. Coverslips were mounted with Fluoromount-G with DAPI (Southern Biotech). Microscopy detection and analysis were performed using a Zeiss Axiovert 200M microscope and the Axiovision software (Zeiss).

### Antibodies

Antibodies used in these studies: Sheep anti-Golgin-84 (gift from Dr. Martin Lowe, University of Manchester), rabbit anti-GM130 (Abcam), rabbit anti-GM130 (Sigma Aldrich), mouse anti-α-tubulin (Sigma Aldrich), rabbit anti-α-tubulin (Abcam), rabbit anti-Golgin-160 (gift from Dr. Carolyn Machamer, Johns Hopkins University), rabbit anti-AKAP450 (Gift from Dr. Mikiko Takahashi, Teikyo Heisei University, Japan), mouse anti-EB1 (BD Biosciences), mouse anti-GAPDH (Santa Cruz), mouse anti-Giantin (gift from Dr. Vivek Malhotra, Center for Genomic Regulation, Barcelona, Spain), mouse anti-Golgin-97 (Invitrogen), mouse anti-polyglutamylated tubulin (Adipogen), rabbit anti-γ-tubulin (Abcam).

### Wound healing assay

A scratch wound, which was introduced into a confluent cell monolayer using a micropipette tip, was imaged at various time points with Zeiss Axiovert 200M microscope (20x objective). Cells were pre-treated with 10μm ROCK inhibitor (Y27632, Calbiochem) or DMSO as solvent control for 12 hours followed by wounding with a micropipette tip. Several images along the wound were taken using Zeiss LSM780 laser scanning microscope and a 10x objective. The gain was adjusted to improve contrast and to aid with visualizing the wound.

### Image analysis

Measurements of wound closure were performed using the Axiovision software (Zeiss). Distance between the Golgi and the centrosome was measured from immunofluorescent images of cells stained with Golgin-84 and γ-tubulin by using the “measure” tool in Axiovert software (Zeiss) and drawing a line from the Golgi edge nearest to the centrosome to the center of the centrosome.

### Flow cytometry analysis

RPE-1 cells were treated with 2mM thymidine (Tocris, Minneapolis) for 17hrs, at which point the cells were washed with PBS and put in fresh media to allow for recovery. After 6 hours in fresh medium, the cells were treated with 10uM RO-3306 (Acros Organics, New Jersey) or 12hrs, and then washed and transferred into fresh media. At the indicated time points post-release, cells were trypsinized and collected into FACs tubes. Cells were pelleted at 300g for 5 minutes at 4°C, then resuspended in 2mL of cold PBS and spun at 300g for 5 minutes at 4°C. Pellets were then fixed in ice cold 70% ethanol, which was added dropwise while vortexing. Resuspended pellets were then stored for further fixation at -20°C for 24hrs. Fixed cells were pelleted at 300g for 5 minutes at 4°C, then resuspended in 20ug/mL propidium iodide and 0.125mg/mL RNase A in cold PBS for 2–4 hours at 4°C. Flow cytometry analysis was performed on the Attune NxT flow cytometer. FlowJo was used to generate the graphs. Singlet nuclei were gated based on scatter properties; 1N and 2N peaks were identified via the YL2-A channel.

### Cell proliferation analysis

Wild-type, KO2 and KO60 cells were seeded at 50,000 cells per well in a 6-well plate. Cells were trypsinized and counted every 24 hours for four days.

### VSV-G-GFP transport assay

Wildtype and GM130 knockout RPE-1 cells were transiently transfected with VSV-Gts045-GFP (gift from Dr. Jennifer Lippincott- Schwartz, National Institute of Health) (Cole *et al*., 1998). At 18 hours post transfection, the cell culture medium was replaced with fresh 10% DMEM supplemented with 25mM HEPES (HyClone). Cells were then transferred to a 41°C incubator supplemented with 5% CO_2_ to allow VSV-G accumulation in the ER. Five hours later, the cells were transferred to permissive temperature (32°C) and incubated for 30 min, 1 h and 2 h. Cells were then fixed with 4% paraformaldehyde and processed for immunofluorescence microscopy.

### Microtubule recovery assay

Cells in cell culture media supplemented with 25mM HEPES were incubated on ice for 40 minutes and shifted to room temperature for 3 minutes. Cells were the rinsed for 40 seconds with microtubule buffer, pH 6.9 (60mM PIPES, 25mM HEPES, 10mM EGTA, 2mM MgCl_2_, 0.25nM Nocodazole, 0.25nM Paclitaxel) and fixed with ice-cold methanol for 7 min.

### Statistical analysis

At least three experiments were done for each experiment. Bar graphs indicate averages of the three experiments, and error bars represent standard deviation between experiments. P values were determined using Student’s t-test and considered significant for p<0.05.

## Results

### GM130 regulates Golgi organization and its association with the centrosome

**Fig 1:**
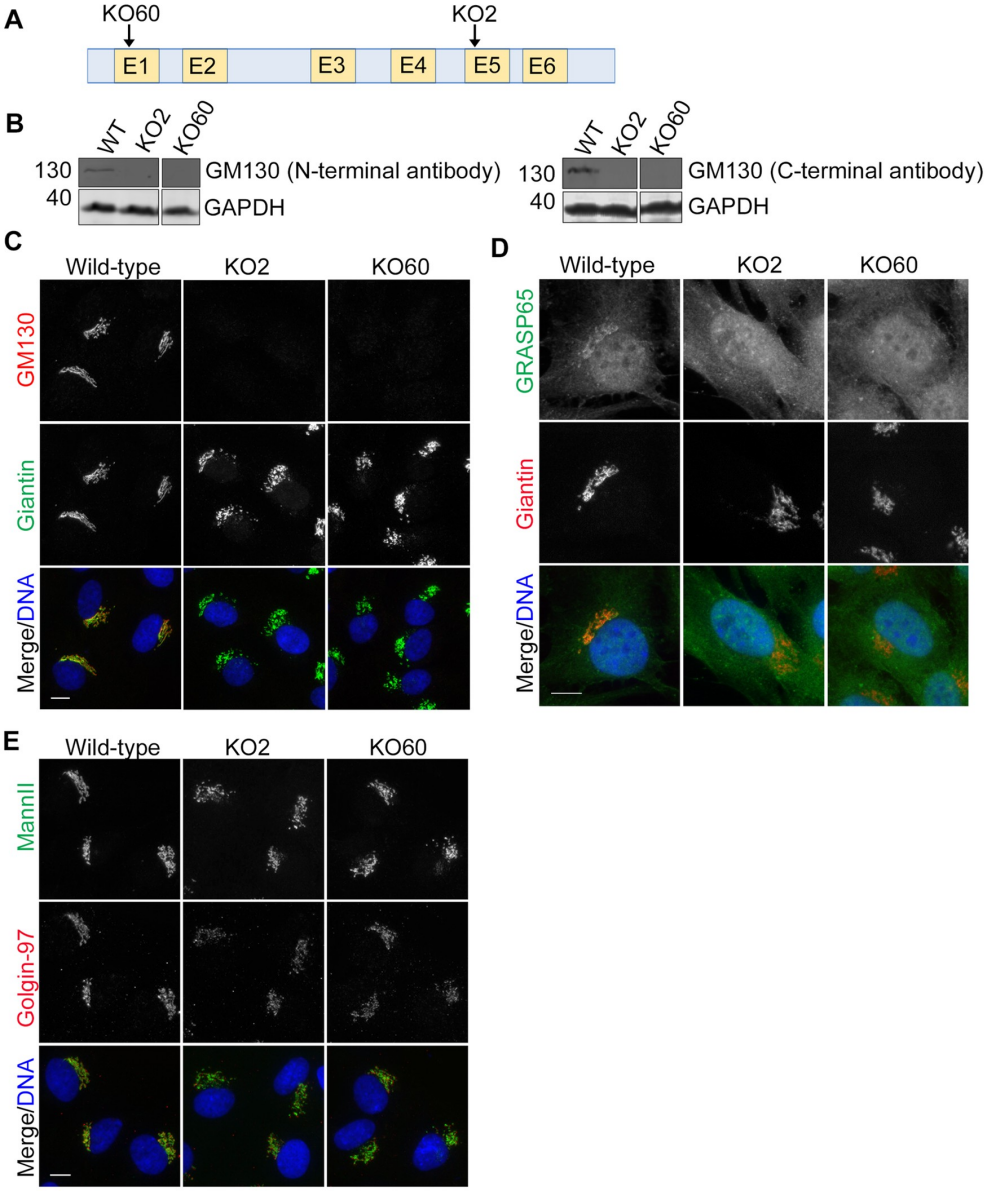
GM130 is required for normal Golgi organization in RPE-1 cells. **A**. Clonal GM130 knockout (KO) cell lines were generated by targeting Cas9 to exon 1 (KO60) or exon 5 (KO2) of *GOLGA2*, the gene encoding for GM130. **B**. Western blotting with an antibody to the GM130 N-terminus (left panel) or C-terminus (right panel). GAPDH served as a loading control. **C—E**. Wild-type and GM130 KO cells were analyzed by immunofluorescence microscopy with antibodies to GM130 and Giantin (**C**.) GRASP65 and Giantin (**D**.) and Mannosidase II and Golgin-97 (**E**.). For each panel, merged images are also shown in which DNA is stained with DAPI. Scale 10μm.

We examined the role of GM130 in Golgi organization in RPE-1 cells. We used a CRISPR-Cas9 knockout approach to disrupt the GM130 locus in immortalized human retinal pigmented epithelial (RPE-1) cells. This untransformed diploid cell line is commonly used for studies of Golgi-nucleated microtubules, directional transport and ciliogenesis. Knockout clone 2 (KO2) was generated by targeting exons 3 and 4 with a set of 4 guide RNAs, which resulted in a premature stop codon due to the loss of 94 nucleotides from the intron between exons 4 and 5 and of one nucleotide from exon five (Fig 1A and S1A Fig). Knockout clone 60 (KO60), produced by targeting a single guide RNA to exon 1, displayed an insertion of 45 base pairs in one allele and a 25 base-pair deletion in the other (Fig 1A and S1B Fig). Loss of the gene product was confirmed for both cell lines by western blot analysis with antibodies to the GM130 N and C-terminus (Fig 1B and S1C Fig) and by immunofluorescence microscopy (Fig 1C). The mislocalization of the known GM130 binding partner GRASP65 from the Golgi to the cytosol further confirmed that GM130 was lost from both knockout cell lines (Fig 1D).

The organization of the Golgi, but not the centrosome, was mildly altered in GM130 KO cell lines. We first monitored overall cell morphology, but did not detect any obvious differences between wild-type and GM130 KO cells (S2 Fig).

We also examined the effects of GM130 loss on cell cycle progression. Cell cycle profiles of asynchronous, propidium iodide-labeled wild-type, GM130 KO2 and KO60 cells were indistinguishable (S2B Fig, left panel). We also synchronized these cells in G2 by thymidine and RO-3306 treatments, followed by RO-3306 washout for 6, 12 or 24 hours to release them synchronously into the cell cycle. We observed cell cycle profiles for KO60 cells that looked similarly to those of their wild-type counterparts (S2B Fig). We were unable to arrest the entire cell population of KO2 cells in G2, which is indicative of a slower rate of cell cycle progression (S2B Fig). However, when we released these cells from the G2 block, they progressed through mitosis into G1, and 24 hours after release, their FACS profile was again indistinguishable from wild-type cells. These FACS results were consistent with cell growth assays (S2C Fig).

We next stained the cells with antibodies to various Golgi proteins to observe Golgi architecture (Fig 1C–1E). In contrast to the wild-type cells with their interconnected Golgi ribbon, the *cis*-Golgi of GM130 KO cells was mildly fragmented, as detected with antibodies to Giantin (Fig 1C and 1D). Fragmentation was also observed for cisternae labeled by the *medial*-Golgi marker Mannosidase II and the *trans*-Golgi protein Golgin-97 (Fig 1E). Interestingly, the Golgi fragments of GM130 KO cells were not dispersed throughout the cell as commonly seen with conditions that induce Golgi fragmentation. Instead, they remained in the perinuclear region of the cell and displayed a stacked organization, as revealed by the presence of *cis*, *medial* and *trans*-Golgi markers adjacent to each other in the same fragment. These results support a role of GM130 in Golgi ribbon formation and are consistent with previous RNAi studies [16].

As abnormalities of the centrosome have been observed in HeLa and U2OS cells depleted of GM130 by RNAi (14,16), we compared the organization of this organelle in wild-type and GM130 KO cells. Antibodies to the centrosomal marker proteins centrin2 and kendrin revealed no evidence of centrosomal abnormalities in GM130 KO cells (S3 Fig), leading us to conclude that centrosome organization of RPE-1 cells is not controlled by GM130. This result also suggests that centrosome organization may be regulated in a cell type-specific manner.

**Fig 2:**
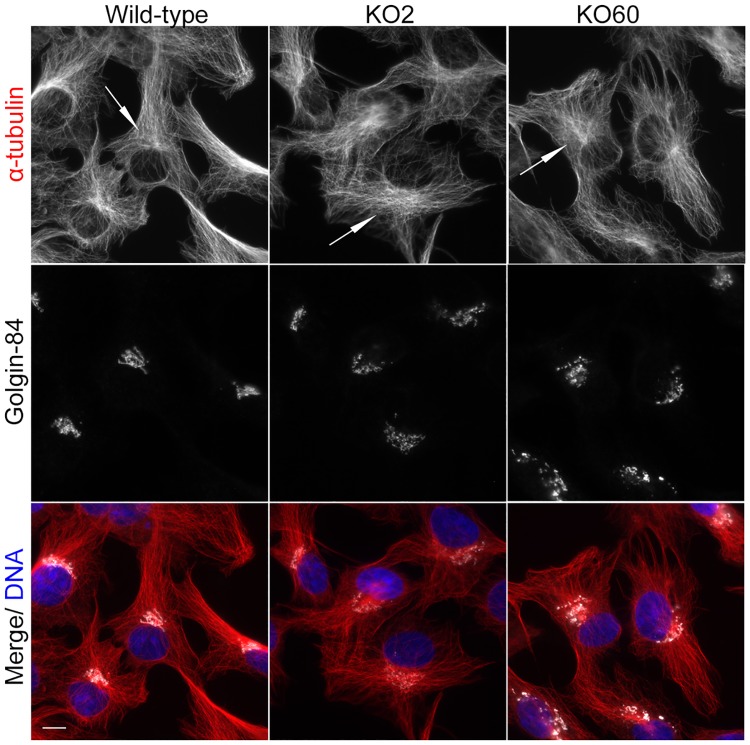
GM130 is not necessary for microtubule organization. Wild-type and GM130 KO cells were stained with antibodies to α-tubulin and Golgin-84 to visualize microtubule organization and Golgi structure, respectively. Merged images in which DNA is stained with DAPI are also shown. The arrows highlight the perinuclear region of these cells. Scale bar, 10μM.

As GM130 has been reported to function in microtubule organization in U2OS cells [14], we compared microtubule organization of wild-type and GM130 KO cells by immunofluorescence microscopy. Overall, microtubules of GM130 KO cells looked fairly normal, with the presence of centrosomal and non-centrosomal microtubules in the perinuclear region (Fig 2). The density of microtubules in the cell center of KO cells was slightly reduced and less focused when compared to their wild-type counterpart (Fig 2, arrows). However, this minor change was unlikely to be caused by a loss of non-centrosomal microtubules because we did not detect the enhanced nucleation of centrosomal microtubules that is typically seen in cells without non-centrosomal microtubules [17,18]. Consistent with this observation, a regrowth assay revealed that KO cells were able to nucleate microtubules from the centrosome as well as non-centrosomal sites in the perinuclear region (S4A Fig, arrows). Antibodies to the plus end-binding protein EB1 or to tubulin acetylation, a post-translational modification characteristic of long lived, stable microtubules, also showed no difference between wildtype and KO cells (S4B and S4C Fig). We conclude that the formation of centrosomal and non-centrosomal microtubules, which both require AKAP450, does not depend on GM130.

**Fig 3:**
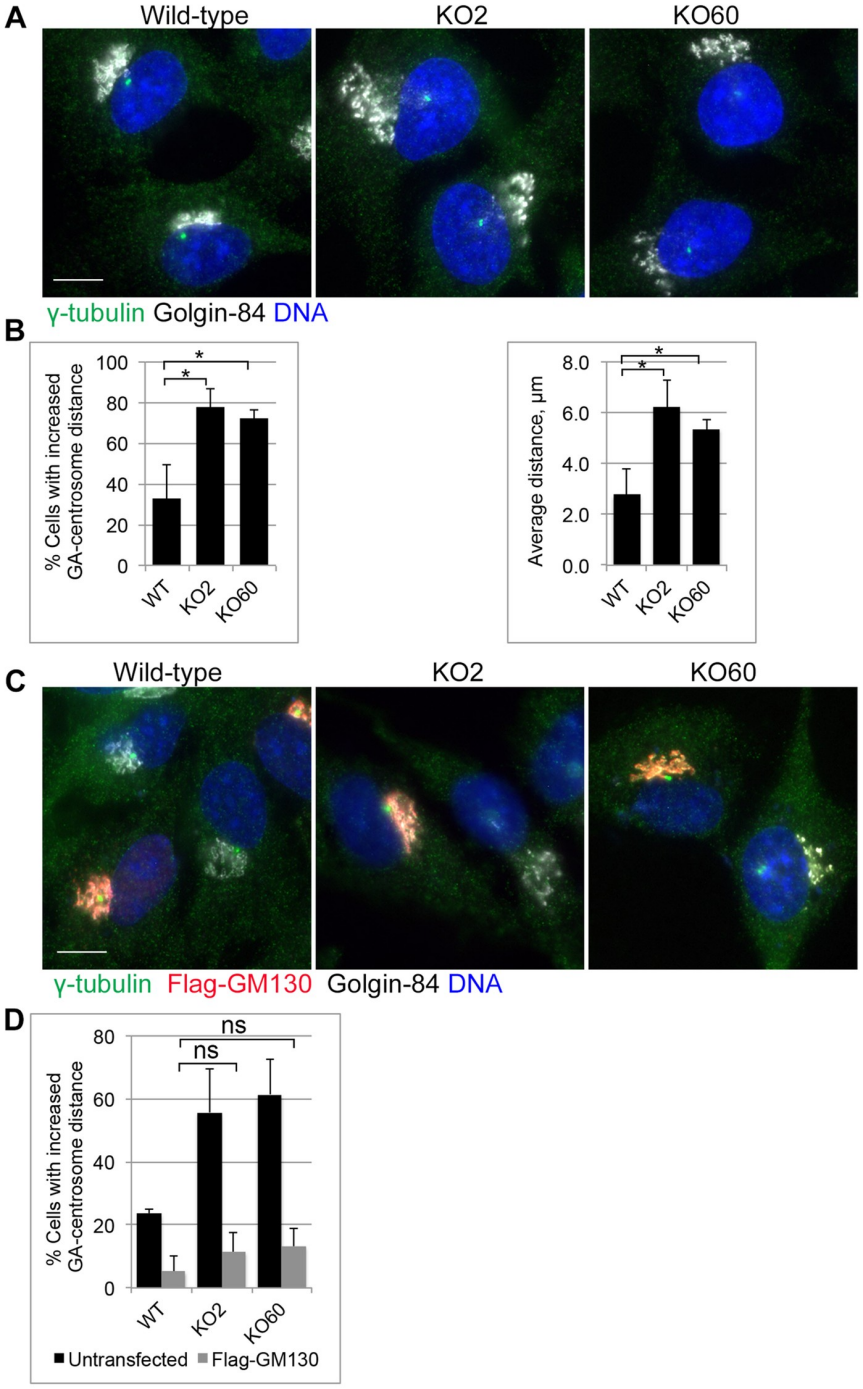
GM130 controls Golgi-centrosome proximity. **A**. Wild-type and GM130 KO cells were stained for γ-tubulin and Golgin-84 to visualize the centrosome and the Golgi, respectively. **B**. Left: Graph showing the percentage of cells with increased Golgi (GA)—centrosome distance. The data is from four independent experiments, with at least 50 cells per condition analyzed. p = 0.006 (KO2) and 0.0014 (KO60). Right: Graph showing the average distance between the centrosome and the nearest Golgi edge. p = 0.0029 (KO2) and 0.0031 (KO60). **C**. Wild-type and GM130 KO cells were transfected with a construct encoding for full length Flag-GM130 and stained for the presence of the Flag tag, γ-tubulin and Golgin-84 to visualize transfected cells, the centrosome and the Golgi, respectively. **D**. Graph showing the percentage of cells (untransfected and transfected) with increased Golgi-centrosome distance. The data is from four independent experiments with at least 40 cells analyzed per experiment. ns = not statistically significant. Scale 10μm.

The absence of GM130 led to separation of Golgi and centrosome. As the GM130 binding partner AKAP450 has been implicated in linking the Golgi and centrosome [12], we examined Golgi and centrosome positioning by co-staining wild-type and GM130 KO cells with antibodies to both organelles (Fig 3A). Careful quantifications revealed that in 70% of wild-type cells, the centrosome was localized within a radius of 2.6μm from the Golgi (Fig 3B). This distance was significantly increased in about 80% of GM130 KO cells (average of 6.2μm and 5.4μm for KO clone 2 and 60, respectively). This phenotype was not due to off-target effects because expression of full-length Flag-tagged GM130 restored the normal distance between these two organelles (Fig 3C and 3D). We conclude that GM130 functions in the maintenance of Golgi-centrosome proximity.

### GM130 may control Golgi-centrosome proximity by recruiting AKAP450 to the Golgi

**Fig 4:**
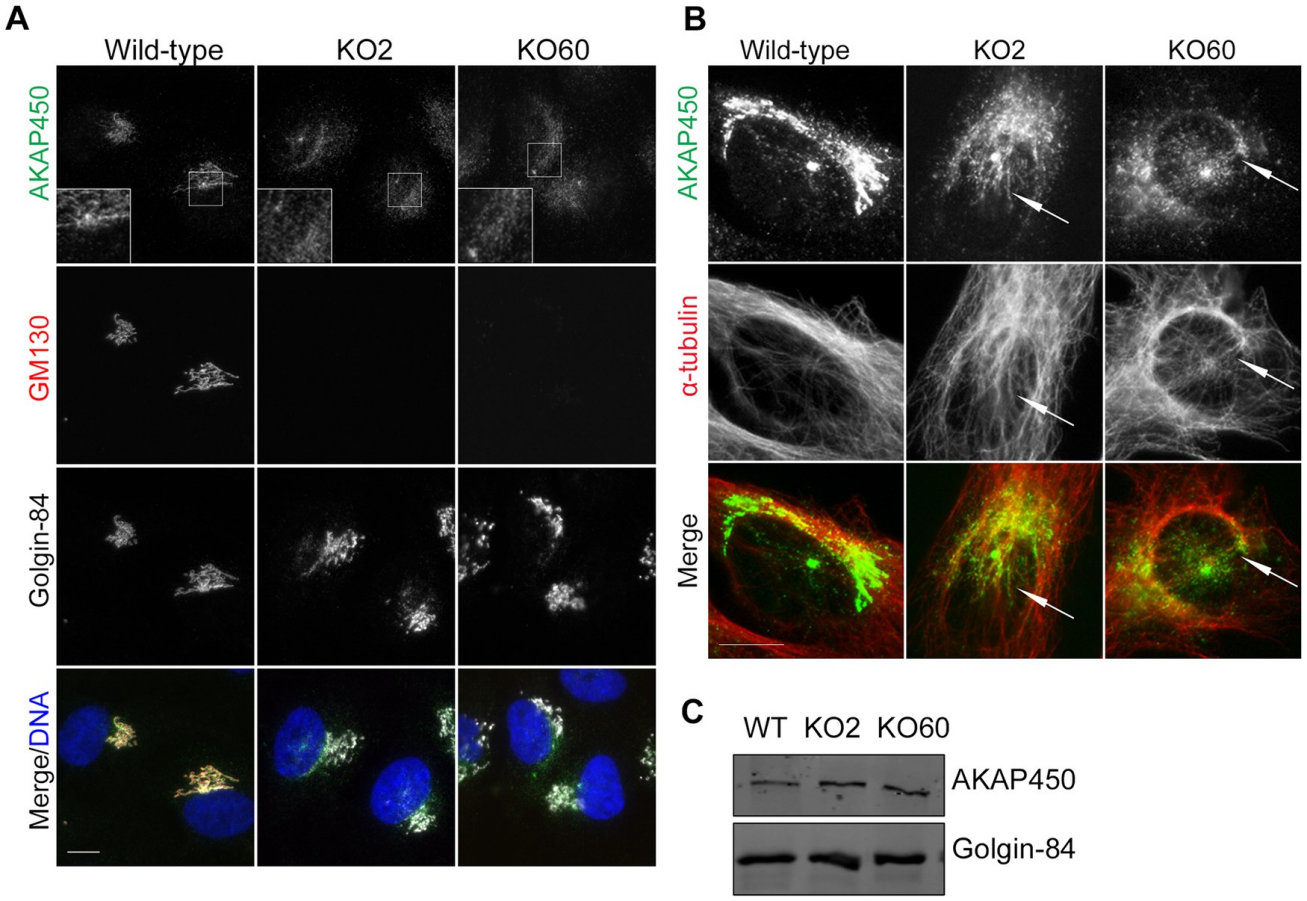
GM130 is necessary for AKAP450 recruitment to the Golgi. **A**. Wild-type and GM130 KO cells were stained with antibodies to AKAP450, Golgin-84 and GM130. Boxes show AKAP450 staining in the area adjacent to Golgi and centrosome **B**. Immunofluorescence analysis of wild-type and KO cells with antibodies to AKAP450 and α-tubulin. Brightness was adjusted to visualize AKAP450 along microtubules. **C**. Western blot analysis of lysates from wild-type and GM130 KO cells with antibodies to AKAP450 and Golgin-84, which was used as a loading control. Scale 10μm.

We assessed the localization of the known GM130 binding partner AKAP450 in GM130-deficient cells. Consistent with reports by Hurtado and colleagues, in wild-type cells, this large scaffolding protein was detected on both the Golgi and the centrosome [19](Fig 4A). However, in GM130 KO cells, although its overall levels were unaffected (Fig 4C), AKAP450 was partially lost from the Golgi [12](Fig 4A). Instead, it was detected in the area between the Golgi and the centrosome (Fig 4A, box), where it colocalized with microtubules (Fig 4B). This specific localization required intact microtubules and was disrupted by ice-induced microtubule depolymerization [20] (S5 Fig). Thus, while GM130 is necessary for AKAP450 anchoring to Golgi membranes, it is dispensable for the transport of AKAP450 to the perinuclear region along microtubules, consistent with previous reports [21].

### Golgi-centrosome proximity is dispensable for protein transport, cell migration and primary cilia formation

**Fig 5:**
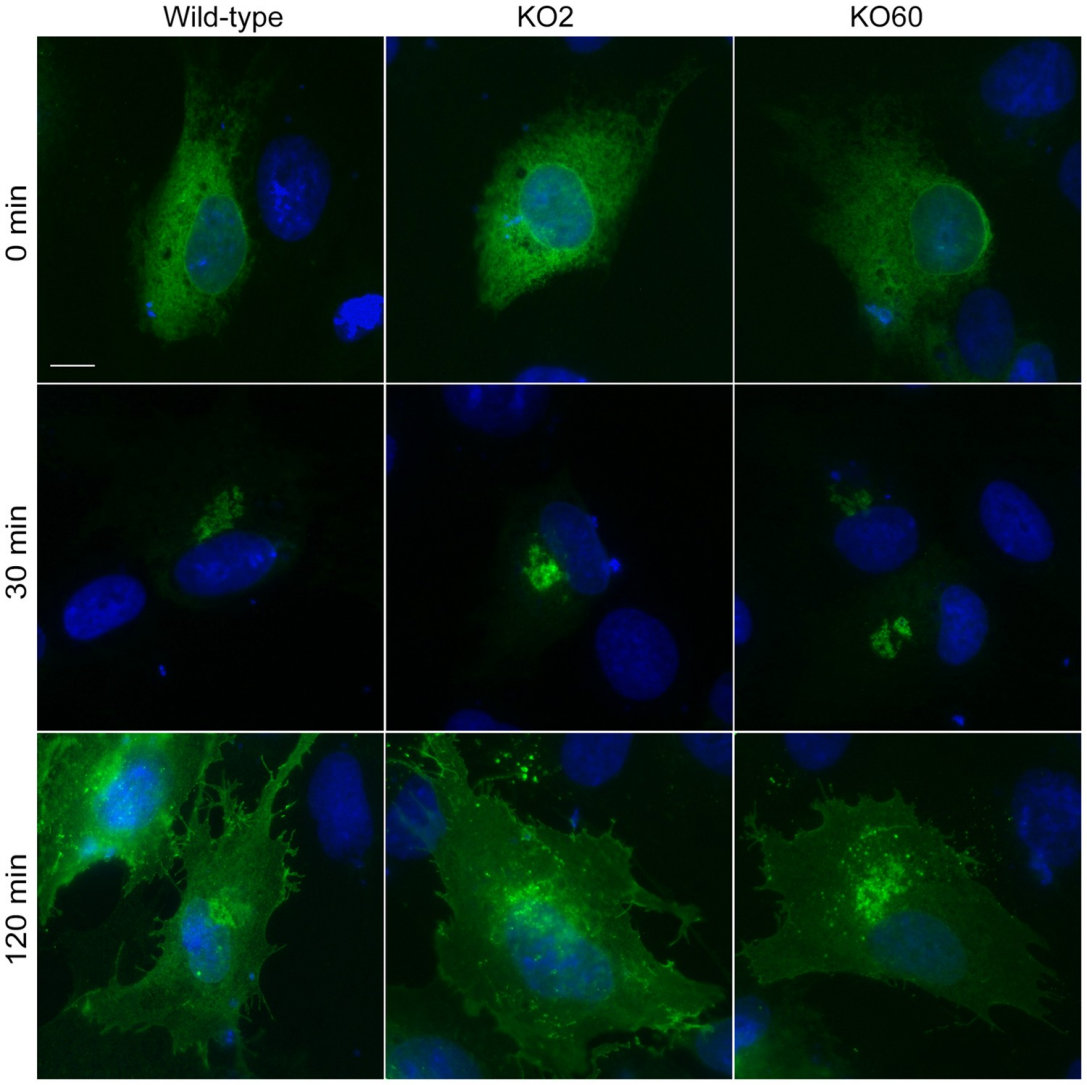
GM130 does not function in protein transport. Wild-type and GM130 KO cells were transfected with tsO45-VSV-G-GFP. 18 hours post transfection, cells were transferred to 42°C for 5 hours to allow VSV-G accumulation in the ER (top panel), followed by a shift to the permissive temperature of 32°C. Cells were fixed after 0 min (top panel), 30 min (middle panel) and 120 min (bottom panel) post transfer. Scale 10μm. Representative immunofluorescence images for each time point are shown, with the GFP signal revealing the localization of the VSV-G protein.

We next compared global protein transport to the cell surface in wild-type and GM130 KO cells, using the well-established VSV-G transport assay. In this assay, a temperature-sensitive mutant of VSV-G (VSV-G tsO45), tagged with GFP, accumulated in the ER at a non-permissive temperature of 41°C for 5 hours. Cells were then shifted to the permissive temperature of 32°C to allow for the synchronous movement of VSV-G through the secretory pathway, which can be detected by fluorescence microscopy. As expected, at non-permissive temperature, VSV-G-GFP was in the ER in wild-type and GM130 KO cells. Upon shifting both cell lines to 32°C, VSV-G was rapid transported to the Golgi and the plasma membrane (Fig 5). These results demonstrate that Golgi-centrosome proximity is not important for protein transport to the cell surface, a finding that is consistent with reports by Hurtado et al. [12].

**Fig 6:**
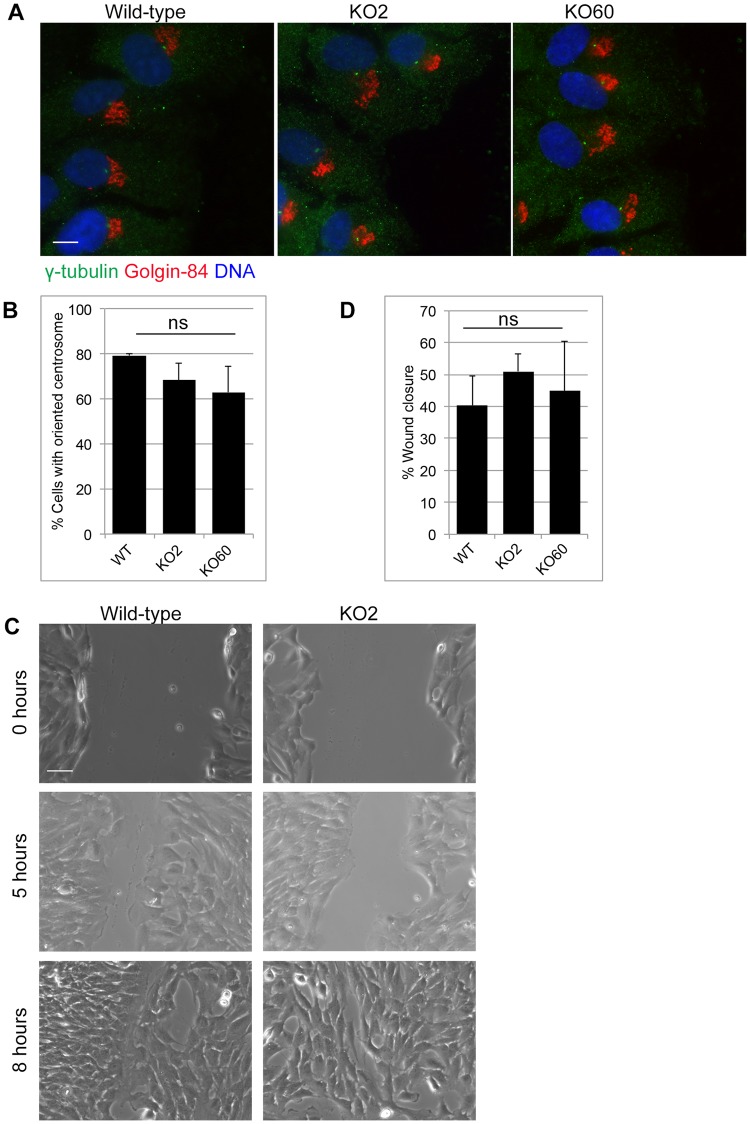
Golgi-centrosome proximity is dispensable for polarization and directional cell migration. A scratch wound was introduced into confluent cell monolayers using a micropipet tip. **A**. Wounded monolayers were fixed 5 hours post wounding and stained for γ-tubulin and Golgin-84. Scale 10μm. **B**. Quantification of the cell polarization assay of Fig 6A. A centrosome was considered oriented if it was found in front of the nucleus, facing the wound. The data is from three independent experiments, with at least 100 cells analyzed per condition. ns = not statistically significant. **C**. Wounds were imaged at 0, 5 and 8 hours after wounding. Representative images for wild-type and KO2 cells are shown. Scale 50μm. **D**. Quantification of wound width for wild-type, KO2 and KO60 monolayers at 0 hours and 5 hours. The data is from three independent experiments, with at least ten measurements at different positions along the wound for each condition.

We next investigated how GM130 KO cells behave in a wound healing assay, which can be used to assess the ability of cells to transport directionally, polarize and migrate [22]. For this assay, we introduced a scratch wound into a monolayer of wild-type or GM130 KO cells and monitored the ability of cells to polarize and close the wound over time. At 5 hours after wounding, the centrosome of 80% of wild-type and 70% GM130 KO cells was detected in front of the nucleus, suggesting a minor, but not statistically significant defect in centrosome reorientation (Fig 6A and 6B). At this same time point, which we chose to avoid wound closure as a result of cell proliferation, we found that wild-type and KO cells had migrated with similar kinetics to close the wound (Fig 6C and 6D). At the 8 hour timepoint, the wound in wild-type and KO cell lines was equally closed (Fig 6C), and this process was sensitive to the ROCK inhibitor Y-27632 (S6 Fig). Our data demonstrates that neither Golgi-centrosome proximity nor GM130 are required for cell polarization or directional cell migration. As cell polarization depends on directional transport, our data also suggests that in RPE-1 cells, GM130 is unlikely to have a role in directional transport.

**Fig 7:**
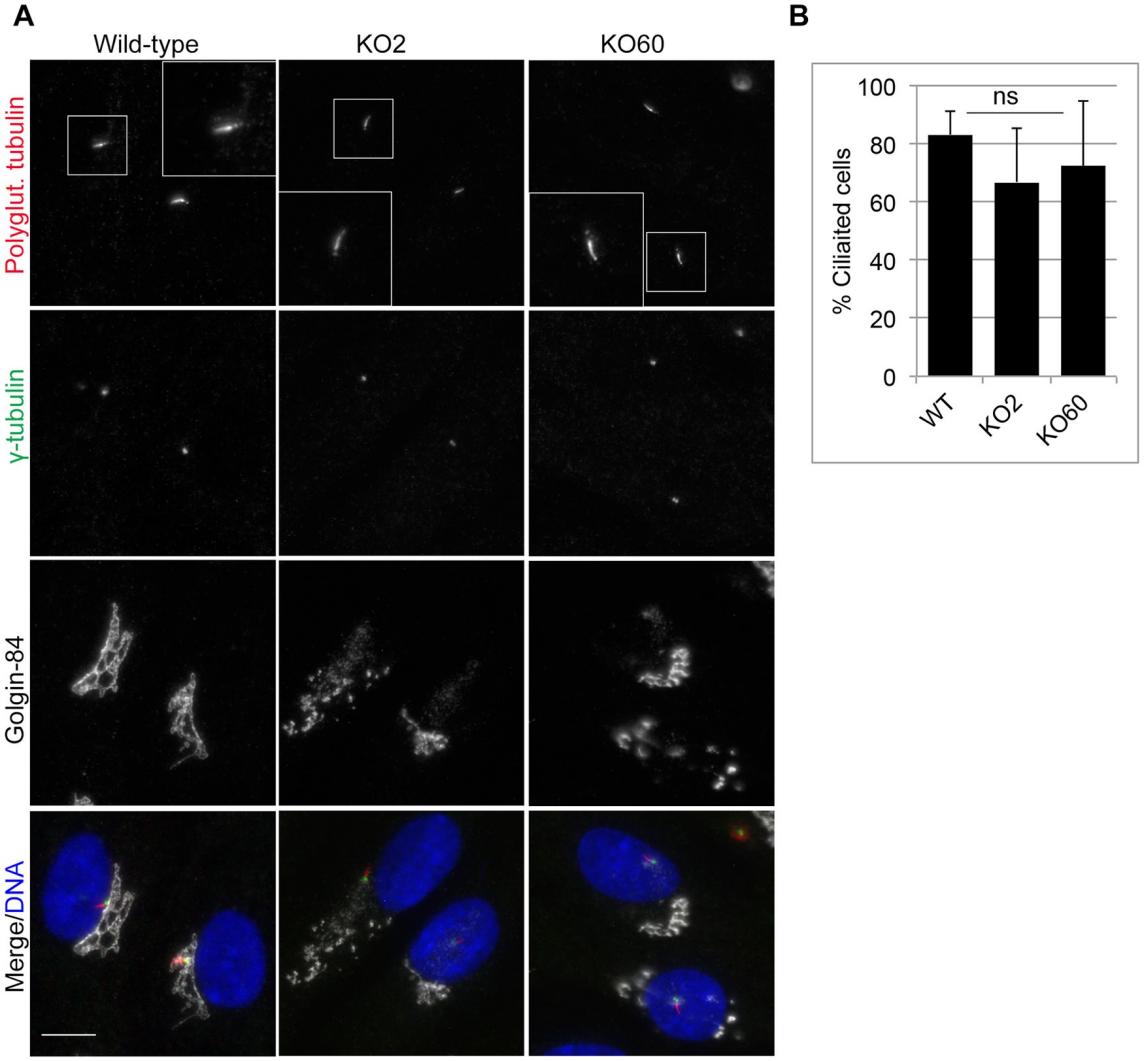
GM130 and Golgi-centrosome proximity are not necessary for ciliogenesis. **A**. Cilia formation was induced by serum withdrawal for 48 hours. Cells were fixed and stained with antibodies to polyglutamylated tubulin, γ-tubulin and Golgin-84 to visualize the ciliary axoneme, the basal body and the Golgi, respectively. Merged images with DNA stained with DAPI are also shown. Scale bar: 10μm. **B**. The percentage of cells with increased Golgi-centrosome distance that were ciliated was quantified. The data is from three independent experiments with at least 50 cells per condition analyzed. ns = not statistically significant.

Finally, we assessed the ability of GM130 KO cells to form primary cilia. As Golgi-centrosome proximity has been implicated in the control of ciliogenesis [12], we compared the percentage of ciliated cells in serum-starved wild-type and GM130 KO cells. For both conditions, primary cilia were seen in about 80% cells (Fig 7A). Further analysis of only those GM130 KO cells with increased Golgi-centrosome distance revealed ciliation in about ~70% of cells (Fig 7B), suggesting that Golgi-centrosome proximity is not necessary for primary cilia formation.

## Discussion

This study aimed at understanding the role of Golgi-centrosome proximity in various cellular processes. Although not present in lower eukaryotes, the unique relationship between these two organelles is a conserved and striking feature of mammalian cells [23,24]. Its functional significance is incompletely understood, mainly because only few experimental manipulations have separated Golgi and centrosome without affecting either Golgi or centrosome organization and function. Here, we present our results with two RPE-1 cell lines in which GM130 was removed by CRISPR-Cas9 and in which the physical proximity between Golgi and centrosome was disrupted.

Our results demonstrate that in RPE-1 cells, Golgi-centrosome proximity is dispensable for cell homeostasis. Prior studies have implicated the physical proximity between these two organelles in the control of higher cellular functions, such as directional protein transport, cell polarization, cell migration and ciliogenesis [12]. However, to our surprise, we found that this specific spatial relationship was dispensable for all of these functions in GM130 KO cells in which Golgi and centrosome were separated. In these GM130 KO cells, protein transport to the cell surface, as measured with the VSV-G transport assay, was normal, which is consistent with reports by Hurtado et al. [12]. Directional migration was normal, with wild-type and GM130 KO cells filling in a wound with similar kinetics. Directional protein transport also appeared unaffected because the directional migration of GM130 KO cells, which depends on directional transport was unaffected [22,25]. Finally, there was no obvious defect in ciliogenesis, although we cannot exclude that kinetics for cilia formation or cilia protein composition and function are impacted by GM130 loss.

While our study found that GM130 is not required for cell cycle progression, our study has not directly addressed the role of Golgi-centrosome proximity in the regulation of mitotic entry. This checkpoint is proposed to sense the ability of Golgi membranes to reorganize at the onset of mitosis, which involves extensive Golgi fragmentation and dispersal and that is a prerequisite for entry into mitosis [26]. It is possible that disconnecting Golgi membranes from the centrosome, as seen in GM130-deficient cells, is sufficient to satisfy this checkpoint, similar to what was reported with nocodazole treatment [26]. Additional experiments are required to better understand this link between organelle structure, positioning and cell cycle progression.

GM130 may control Golgi ribbon formation and Golgi-centrosome proximity through AKAP450. Prior studies have shown that AKAP450 controls the nucleation of non-centrosomal microtubules from the Golgi by recruiting γ-TuRC as well as the microtubule nucleation modulators CDK5RAP2 and myomegalin to the Golgi [18,27–29]. However, the ability of this protein to nucleate microtubules was independent of its location and was still observed when AKAP450 was mislocalized to ER exit sites [21]. In GM130 KO cells, AKAP450 was mislocalized from the Golgi to the pericentriolar region, which it reaches via continuous dynein and microtubule-dependent transport [20]. Therefore, in the absence of GM130, AKAP450-mediated microtubule nucleation is shifted from the Golgi to the perinuclear region. This novel set of perinuclear-derived microtubules is able to partially compensate for those microtubules that are normally nucleated at the Golgi, promoting, for example, directional transport and cell migration. However, it was unable to induce the formation and maintenance of the Golgi ribbon, which strictly requires Golgi-nucleated microtubules [30,31]. Loss of AKAP450 from the Golgi may also interfere with bringing Golgi and centrosome in physical proximity. Consistent with this idea, disrupting AKAP450 function through expression of the N-terminus of AKAP450, which also binds GM130 and dynein, phenocopied the loss of Golgi-centrosome proximity of GM130 KO cells [12]. However, further investigation will be necessary to dissect these specific molecular interactions and their contributions to the Golgi-centrosome network.

How do our results compare to other studies that investigated either the role of GM130 itself or Golgi-centrosome proximity? Our GM130 knockout RPE-1 cells recapitulated the reported roles of GM130 in Golgi ribbon formation [16] and in the nucleation of microtubules at the Golgi [21]. Our data is also consistent with a function for GM130 in linking Golgi and centrosome, as reported for GM130-depleted U2OS and HeLa cells, as well as for neurons from GM130 knockout mice [14,15]. However, our study also revealed obvious inconsistencies that may be due to differences in cell lines or experimental approach. For example, while GM130 was found to be required for migration of A549 lung epithelial cells [14], RPE-1 cells migrated normally in its absence. GM130 is proposed to control cell migration through the activation of YSK1 and Cdc42 [32–34], but it is unclear how these regulatory factors are activated in the absence of GM130-deficient RPE-1 cells. Another inconsistency lies in the role of Golgi-centrosome proximity. Hurtado et al. found that RPE-1 cells with separated Golgi and centrosome were defective in directional protein transport, cell migration and ciliogenesis [12]. However, our GM130 KO cells did not show a defect in any of these processes. While both studies used RPE-1 cells, we cannot exclude the possibility that our experimental strategy selected for clones that are able to compensate for the lack of GM130, which may be a shortcoming of the CRISPR/Cas9 approach. Alternatively, it is possible that the previously used siRNAs produced off-target effects.

In summary, we show that in RPE-1 cells the close proximity between Golgi and centrosome is dispensable for cellular functions, such as migration, protein transport and primary cilia formation, which challenges our current understanding of the significance of this striking feature of mammalian cells.

## Supporting information

S1 Fig Establishment of GM130 KO RPE-1 cell lines. Sequencing of wild-type and GM130 KO clones revealed a loss of 94 bases in clone 2 (**A**), an insertion of 45 bases in one allele of clone 60 and a deletion of 25 bases in the other allele (**B**). **C**. Full image of the blots shown in Fig 1B.
(TIF)

S2 Fig The absence of GM130 does not affect cell morphology and proliferation. **(A)** Bright field images of wild-type and GM130 KO cells. Scale 20μm. **(B)** Asynchronous populations of wild-type and GM130 KO clones were arrested in G2 by treatment with thymidine and RO-3306 as described in the materials and methods. Cells were then allowed to re-enter the cell cycle and fixed 0, 6, 12 and 24 hours post release, stained with propidium iodide and analyzed by flow cytometry. The Y-axis shows the number of cells, the X-axis the DNA content based on propidium iodide staining. **(C)** Wild-type, KO2 and KO60 cells were seeded at 50,000 cells per well in a 6-well plate. The number of cells/well following trypisinization is shown at the indicated time point.
(TIF)

S3 Fig GM130 is not necessary for centrosome structure maintenance. Wild-type and GM130 KO cells were stained with antibodies against centrin2 and Kendrin to visualize centrosome structure. Magnified images are shown in the boxes. Scale 10μm.
(TIF)

S4 Fig GM130 is not necessary for microtubule organization. (**A**) Wild-type and GM130 KO cells were incubated on ice for 40 minutes to depolymerize microtubules. Cells were then transferred to room temperature for 3 minutes to allow microtubule regrowth. Cells were stained with antibodies against α-tubulin and AKAP450. Arrows point to microtubules growing from non-centrosomal, perinuclear sites. Scale 10μm. (**B**) Wild-type and GM130 KO cells were stained with antibodies to EB1 to visualize microtubule plus ends. Scale 10μm or (**C**) with antibodies against acetylated tubulin to determine organization of stable microtubules. Scale 10μm.
(TIF)

S5 Fig GM130 is necessary for microtubule-dependent AKAP450 recruitment to the Golgi. **(A**) Wild-type and GM130 KO cells were stained with antibodies to AKAP450, Golgin-84 and α-tubulin to visualize AKAP450 localization in relationship to the Golgi and microtubules. (**B**) Cells were placed on ice for 40 minutes to depolymerize microtubules and stained as in (**A**) Scale 10μm.
(TIF)

S6 Fig GM130 is not necessary for cell migration. GM130 KO2 and KO60 cells were treated with either 10μM Y-27632 or DMSO as a negative control for 12 hours. Cell monolayers were wounded using a micropipette tip, followed by imaging at various positions along the wound at 0 hours, 5 hours and 8 hours post wounding. Representative images of wounds are shown. Scale 100μm.
(TIF)
