# Supplementary figures and images for: Function of Golgi-centrosome proximity in RPE-1 cells
Source: PLoS One. 2019 Apr 15;14(4):e0215215. doi: 10.1371/journal.pone.0215215 (PMC6464164; doi:10.1371/journal.pone.0215215)

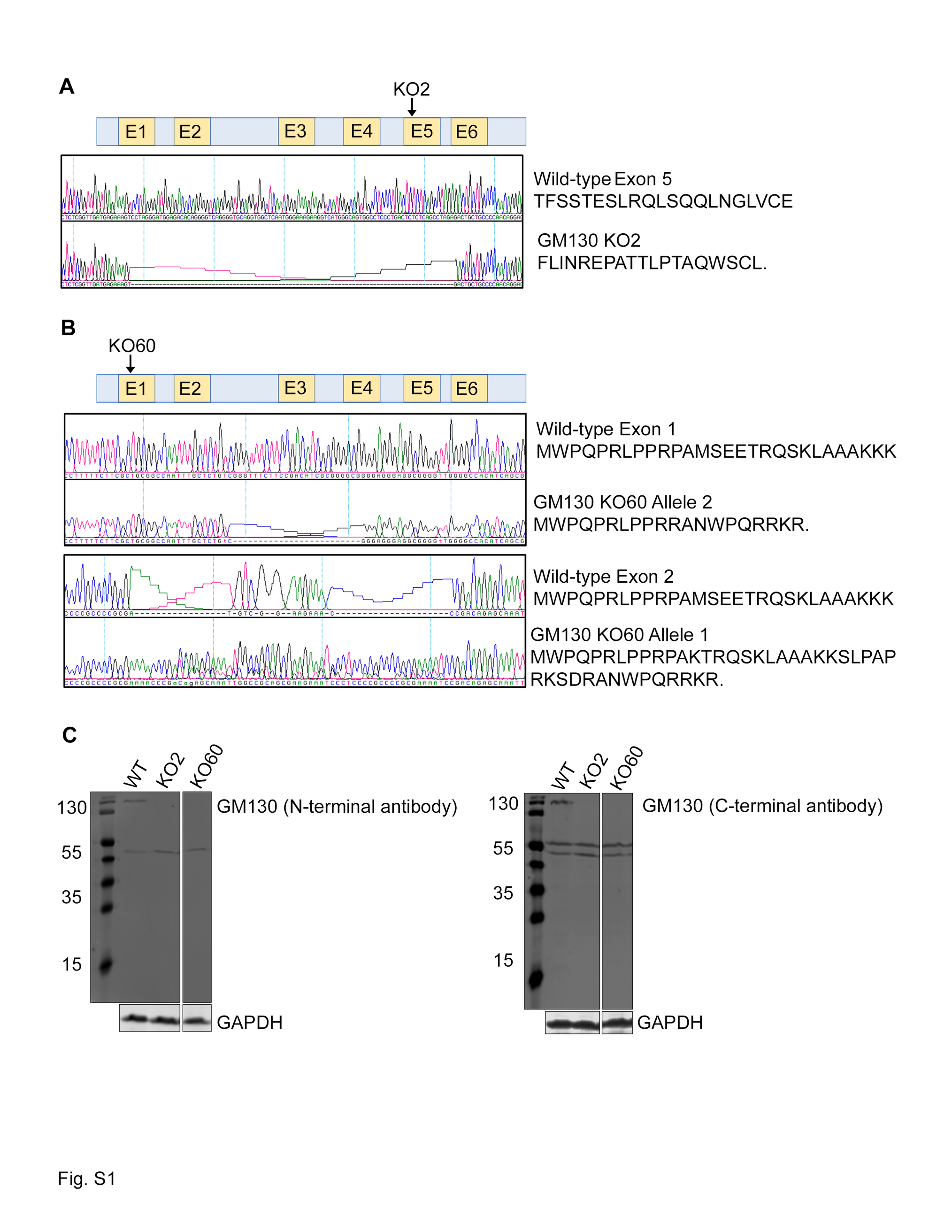

Supplement: S1 Fig — Sequencing of wild-type and GM130 KO clones revealed a loss of 94 bases in clone 2 (A), an insertion of 45 bases in one allele of clone 60 and a deletion of 25 bases in the other allele (B). C. Full image of the blots shown in Fig 1B. (TIF) [file pone.0215215.s001.tif]

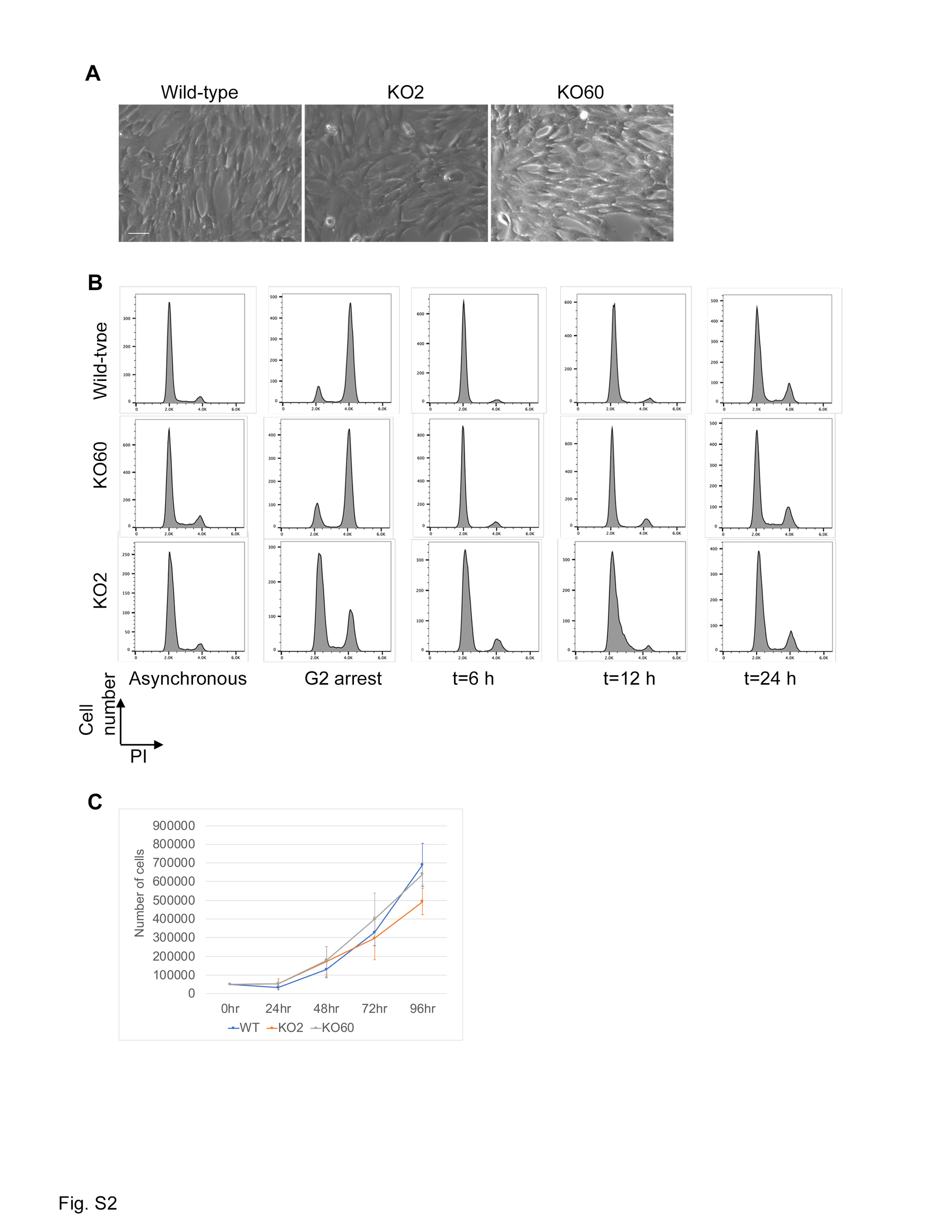

Supplement: S2 Fig — (A) Bright field images of wild-type and GM130 KO cells. Scale 20μm. (B) Asynchronous populations of wild-type and GM130 KO clones were arrested in G2 by treatment with thymidine and RO-3306 as described in the materials and methods. Cells were then allowed to re-enter the cell cycle and fixed 0, 6, 12 and 24 hours post release, stained with propidium iodide and analyzed by flow cytometry. The Y-axis shows the number of cells, the X-axis the DNA content based on propidium iodide staining. (C) Wild-type, KO2 and KO60 cells were seeded at 50,000 cells per well in a 6-well plate. The number of cells/well following trypisinization is shown at the indicated time point. (TIF) [file pone.0215215.s002.tif]

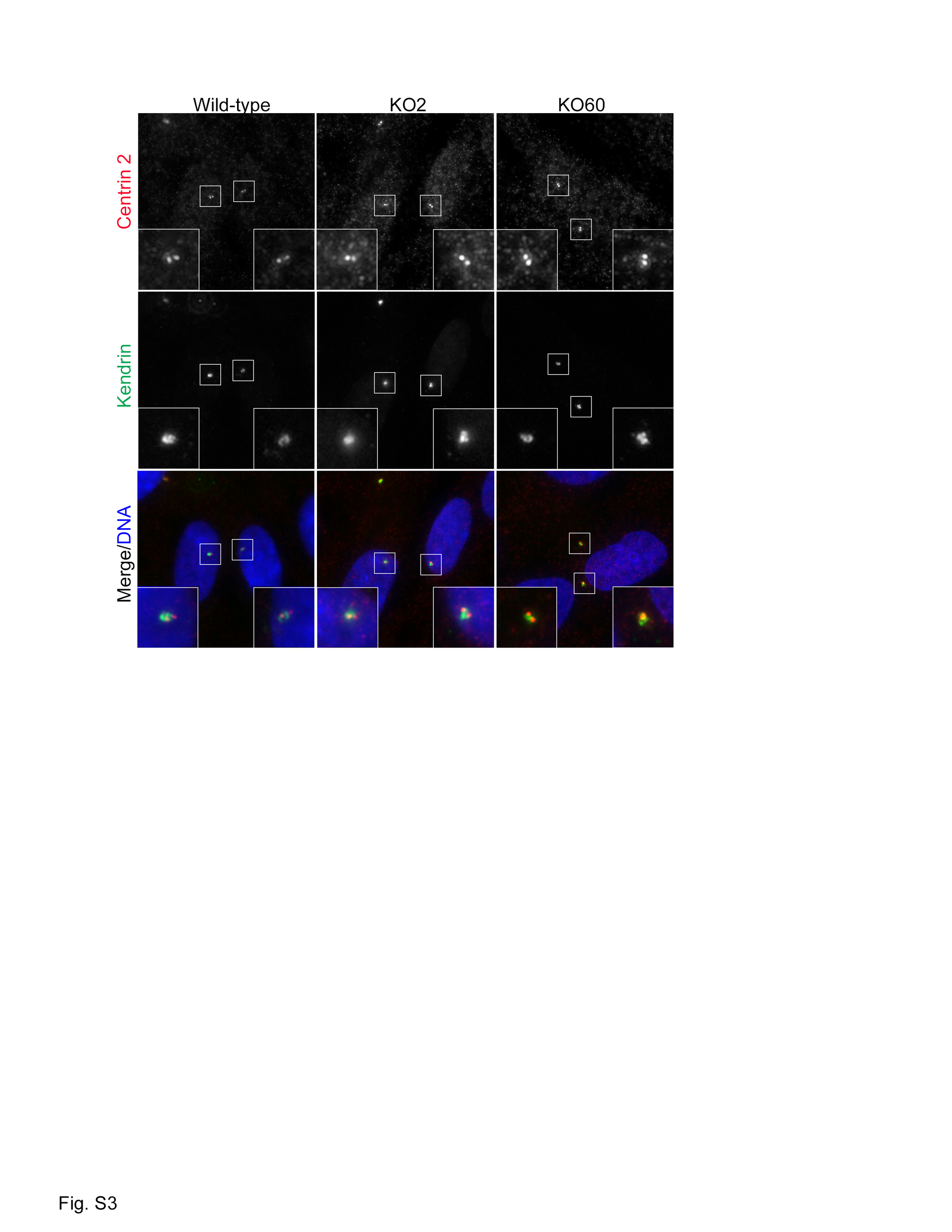

Supplement: S3 Fig — Wild-type and GM130 KO cells were stained with antibodies against centrin2 and Kendrin to visualize centrosome structure. Magnified images are shown in the boxes. Scale 10μm. (TIF) [file pone.0215215.s003.tif]

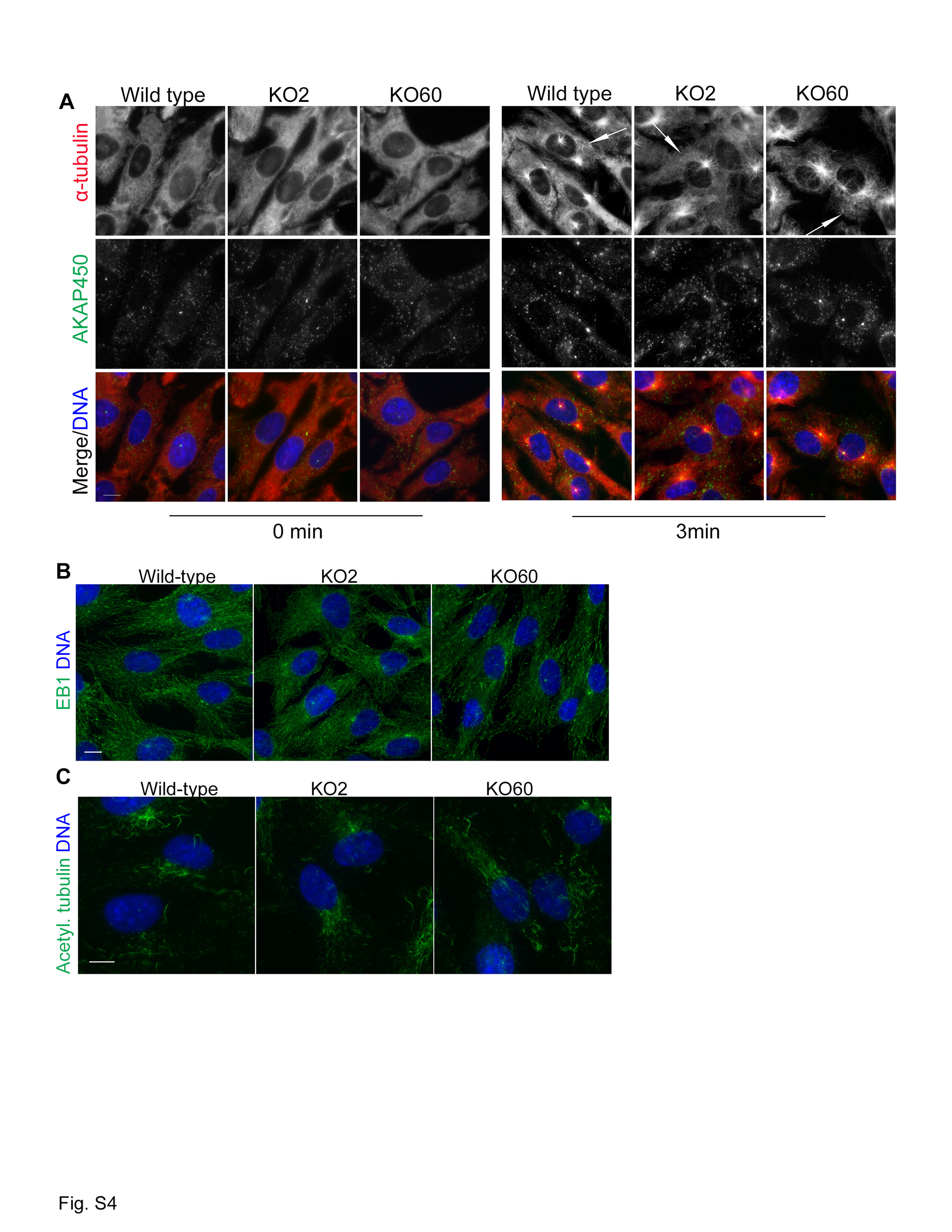

Supplement: S4 Fig — (A) Wild-type and GM130 KO cells were incubated on ice for 40 minutes to depolymerize microtubules. Cells were then transferred to room temperature for 3 minutes to allow microtubule regrowth. Cells were stained with antibodies against α-tubulin and AKAP450. Arrows point to microtubules growing from non-centrosomal, perinuclear sites. Scale 10μm. (B) Wild-type and GM130 KO cells were stained with antibodies to EB1 to visualize microtubule plus ends. Scale 10μm or (C) with antibodies against acetylated tubulin to determine organization of stable microtubules. Scale 10μm. (TIF) [file pone.0215215.s004.tif]

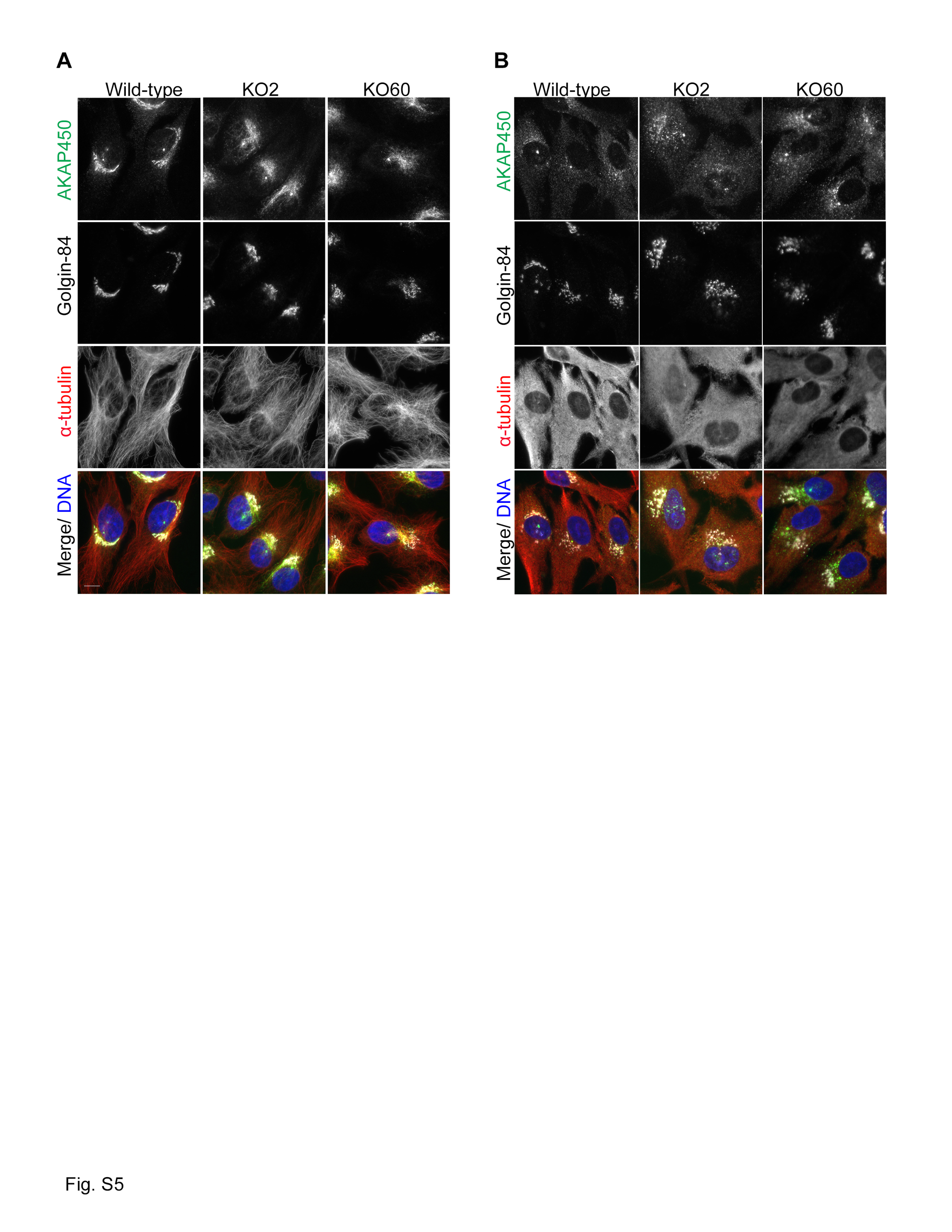

Supplement: S5 Fig — (A) Wild-type and GM130 KO cells were stained with antibodies to AKAP450, Golgin-84 and α-tubulin to visualize AKAP450 localization in relationship to the Golgi and microtubules. (B) Cells were placed on ice for 40 minutes to depolymerize microtubules and stained as in (A) Scale 10μm. (TIF) [file pone.0215215.s005.tif]

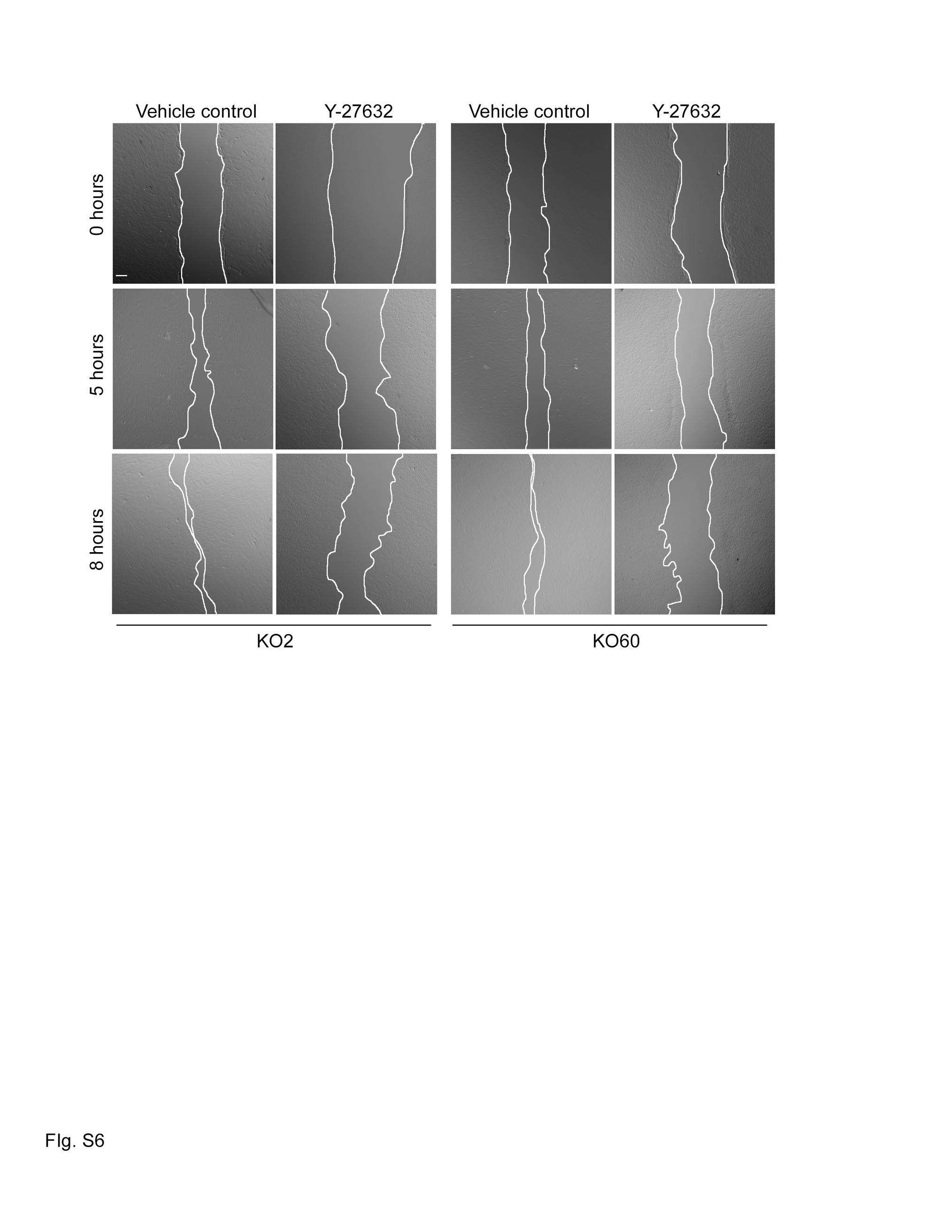

Supplement: S6 Fig — GM130 KO2 and KO60 cells were treated with either 10μM Y-27632 or DMSO as a negative control for 12 hours. Cell monolayers were wounded using a micropipette tip, followed by imaging at various positions along the wound at 0 hours, 5 hours and 8 hours post wounding. Representative images of wounds are shown. Scale 100μm. (TIF) [file pone.0215215.s006.tif]
